# Use of synthetic communities to study microbial ecology of the gut

**DOI:** 10.20517/mrr.2021.11

**Published:** 2022-01-20

**Authors:** Maryse Berkhout, Erwin Zoetendal, Caroline Plugge, Clara Belzer

**Affiliations:** Laboratory of Microbiology, Wageningen University & Research, Wageningen 6708 WE, Gelderland, The Netherland.

**Keywords:** Intestinal microbiome, synthetic microbial communities, *in vitro *fermentation, complex microbial ecosystem

## Abstract

The application of *in vitro *synthetic microbial communities is an excellent approach to model the ecological interactions between microbes in the human gastrointestinal tract. Although DNA-based studies have provided a wealth of information, they do not consider the ecological properties of the human gut microbiota. Ecological interactions between gut microbes of interest can be studied by applying synthetic communities. This review describes the considerations that should be taken into account when constructing a synthetic community by discussing example research questions that can be answered by using a synthetic microbial community, the choice of microbial species, the growth conditions, possible reactor setups, and the parameters to analyze.

## INTRODUCTION

The human intestine is home to a complex microbial ecosystem that plays an important role in the conversion of indigested food components and in defending our body against unwanted intruders. Our knowledge on the microbiota, i.e., the collective number of microbes in the intestine, in relation to our health and diet has dramatically increased. This is in part due to explosive technological developments in high-throughput DNA-targeted approaches such as next generation sequencing in the past decades^[1,2]^. Nowadays, studies that include 16S rRNA gene or metagenome profiles from hundreds to thousands of subjects are no longer an exception. Among other observations, these studies have collectively revealed that the microbiota composition differs between individuals, thereby confirming and extending the observations of individuality of the microbiota based on 16S rRNA gene fingerprinting^[3]^. In addition, the microbiota composition shows from birth a succession towards a stable diverse adult microbiota composition, which subsequently declines on average in diversity during aging. Remarkably, metagenomics analyses have demonstrated that the functions encoded by the microbiota show high similarities between individuals, which seems to contrast with the individuality at 16S rRNA gene observations^[4]^. Combined, these observations indicate functional redundancy among various intestinal microbial taxa.

Although these extensive “DNA-based” studies have collectively provided a wealth of information, even within the most deeply phenotyped cohorts, only a small fraction of the compositional diversity within the intestinal microbiota between humans can be explained by factors such as diet and host genetics and physiology^[1,2,5]^. A recent comment in Nature identifies that many fundamental questions remain unanswered^[6]^. Part of this can be explained by the vast (micro)biological variation between humans and the fact that most microbiota studies rely on snapshot cross-sectional analyses. For example, a recent study demonstrated that two Dutch pre-diabetic cohorts with nearly identical selection criteria for recruitment showed drastic microbiota differences at baseline^[7]^. This exemplifies that any cross-sectional comparison between groups of subjects will likely provide microbiota compositional differences, and that cross-sectional comparisons alone are too limited to understand the biological differences underlying the observed variation in composition. In addition, it is very likely that the variation in microbiota composition between individuals is due to the functional redundancy between microbes in combination with the versatility of microbes, which easily change their metabolism depending on the environmental conditions. Indeed, intervention studies targeting the microbiota frequently show a minor impact on microbial composition but a large impact on microbiota activity, suggesting that microbes change their activity as a response to a change in their environment^[8,9]^. This important ecological characteristic of microbes is not reflected in data generated by DNA-based approaches. Hence, there is a need for microbial ecological approaches that take functional redundancy and versatility of microbes into consideration in order to increase our understanding of the intestinal microbiota. This can be done by *in vitro *incubation studies in which the activity of microbes can be assessed and controlled.

Numerous *in vitro *models to study the microbiota in the intestine have been developed and validated. These include, among others, “simple” batch incubations, fermenter systems, complex models simulating different parts of the intestine, and dedicated systems to study host-microbe interactions^[10]^. These *in vitro *models can be inoculated with fecal specimens or a selected set of microbes also called a synthetic community. The use of fecal specimens as inoculum has the benefit that the *in vitro *system uses the original set of microbes from an individual and thereby reflects the closest approximation of their respective microbiota. Several *in vitro *model studies using fecal inocula have already reflected that the microbiome is highly personalized and influenced by other sources of variation, such as age of the host and intestinal location that are observed *in vivo*^[11-13]^. However, the intestine can only be approximated in any *in vitro *model, and it is evident that the choice of media and conditions results in a selection of microbes that are best adapted to the respective conditions. The use of synthetic communities has the benefit that microbes can be selected as representatives of certain functions within the ecosystem, and that is fruitful for obtaining mechanistic insights into their activity within the ecosystem and how microbes interact with each other. This review focuses on synthetic communities of the human intestine and how these can be used to study the human intestinal ecosystem.

## HOW TO CONSTRUCT A SYNTHETIC MICROBIAL COMMUNITY

To construct a synthetic microbial community, it is key to begin with well-defined research questions and hypotheses. This simplifies the experimental design, which includes but is not limited to: (1) the choice of representative microbes; (2) defining the growth conditions; (3) selecting the reactor setup; and (4) deciding the parameters to analyze [Table 1].

**Table 1:** Considerations for constructing a synthetic microbial community

Choice of representative microbes 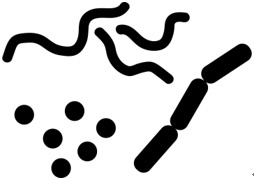	• Natural growth environment • Microbial activities • Genome sequenced • Culturable • *In vivo *presence and abundance • Compatibility with other microbes of interest • Growth rate *in vitro* • Number of species
Defining the growth conditions 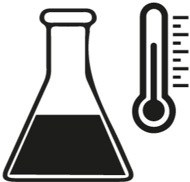	• Energy, carbon, and nitrogen source(s) • Nutrients • pH • Redox conditions: oxic/anoxic • Timing of nutrient supply
Selecting the reactor setup 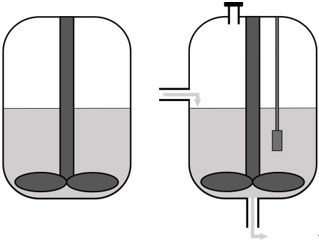	• Batch • Continuous bioreactor • Mini-bioreactor • Complex model
Parameters to analyze 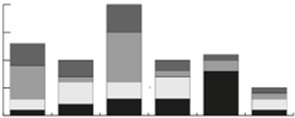	• Microbial composition and relative abundance • Transcriptome • Proteome • Metabolome • Tracing substrate • Visualization

### The choice of representative microbes

The choice of which microbes to include in the synthetic community depends on the research question. For example, a synthetic community can be a model of a certain environment, i.e., a specific region of the gut (small or large intestine, lumen, or mucosal layer); a community that performs a certain action, such as the degradation of dietary glycans; a trophic interaction, e.g., cross-feeding on another species’ metabolites; or a disease mechanism, for instance overgrowth of an opportunistic pathogen. Species that are involved in the environment and action of interest can be selected based on whether their genomes have been sequenced, their functional roles in the gut, and their respective *in vivo *presence and abundance. A sequenced genome is essential for the downstream analyses of the synthetic community, and knowledge about function, presence, and abundance allows a solid design of a community that best represents the *in vivo *situation of interest. There are multiple datasets available that can aid in the selection of gut species of interest. These include, among others, the Human Microbiome Project, gutMDisorder, MetaHit, and the Unified Human Gastrointestinal Genome^[11,14-16]^. An overview of cultured species can be found in databases such as Bac*Dive*^[17]^. Furthermore, it is vital to test if the anticipated microbial species for the synthetic community can grow together. For example, the chosen medium should allow microbes to grow under conditions of similar pH and temperature, and it should be considered that there can be production of metabolites that are toxic to some microbial community members. Another consideration is growth speed, which could lead to an advantage for faster-growing species over slower-growing species. This can be accounted for by adjusting the dilution rate accordingly when using a chemostat or by growing the synthetic community in (fed-)batch. Finally, the number of species that are to be included in the synthetic microbial community should be defined. The more complex the synthetic community is, the more interactions can be modeled. However, the smaller the community is, the easier these interactions between the microbes can be monitored. A functionally minimal community could be defined, where all functions of interest are covered, so that each microbial species is a representative of a certain function and thus performs its own role in the community. It should, however, be recognized that the presence of a certain function in an organism’s genome does not guarantee expression of this function under all experimental conditions. Therefore, setting up a functionally minimal community requires careful experimental design that includes validating the expression of the function of interest. Another approach is defining a community with functional redundancy, which may lead to a more stable community, but it could also induce competition between microbes with a similar functional niche. Furthermore, the design of the synthetic community can be model guided, for example when the goal is to achieve an optimal functional outcome^[18]^. Tools such as gapseq, which is applied for metabolic pathway and network reconstruction, can be useful for designing a synthetic microbial community, as well as for optimization during the experimental phase^[19]^.

### Defining the growth conditions

The conditions in which the synthetic microbial community will grow must be defined. For example, for modeling the gut microbiota in the colon, the culture environment should be anoxic, which allows growth of gut anaerobes. This can be achieved by gas exchanging the head space of the reactor or flask that is used to the desired gas phase and including a suitable reducing agent in the medium. Furthermore, the medium should contain a buffer or continuous automatic pH control, which should warrant a stable pH during experiments to ensure reproducibility. The pH should be set to reflect the environment that is modeled in the synthetic community and allow the selected species to thrive. Another consideration is the choice of the medium to use for the synthetic community. There are complex media available that mimic a selected part of the intestinal tract, such as simulated-ileal-environment medium and simulated-colonic-environment medium^[20,21]^. Alternatively, a basal-defined medium creates a more controlled system^[22]^. Next, the sources of energy, carbon, and nitrogen can be selected. This choice largely depends on the research questions because there are many potential substrates in the gut, such as dietary carbohydrates and proteins, host-derived mucin glycans in general, or human milk oligosaccharides in the infant gut. Depending on the location in the gut, the function of interest, and the species present in the synthetic microbial community, one or multiple of these substrates can be added to the medium. To incorporate dietary components as nutrients for the synthetic community, it should be considered that a diet is highly variable, in terms of both timing and composition. *In vivo*, dietary components are not constantly available to the gut microbiota. Therefore, dietary nutrients can be added to the synthetic community at regular intervals, instead of providing a constant supply^[23]^. Furthermore, the composition of dietary nutrients should be considered carefully, as there is large inter- and intra-individual variation in food intake^[24]^. Diet greatly influences the composition of the gut microbiota, leading to variations in its composition, for example by geographical location; by varying intakes of fibers, fat, protein, and carbohydrates; and by a predominantly vegetarian or carnivorous diet^[25]^. A synthetic community can be constructed with the aim to cover as many (major) functions of the *in vivo *human gut microbiota as possible. For this approach, a complex medium is required. For example, one experiment consisted of a bioreactor which was provided with a constant flow of mucin and acetate, and the dietary components pectin, xylan, starch, and inulin were added three times per day to mimic a human diet to construct a minimal microbiome that covered key ecological and metabolic properties of the human gut microbiota^[23]^. Alternatively, the focus of the synthetic community can be on a single function of the human gut microbiota. For example, mucin degradation was studied by growing mucin-degrading bacteria together with their syntrophic partners in a medium containing mucin as the sole carbon source^[26,27]^.

### Selecting the reactor setup

The next consideration is the *in vitro *system in which the synthetic microbial community will grow. The community can be grown in batch in flasks, which is a straightforward method to study interactions in a simple microbial community under controlled conditions. However, batch cultivation leads to quick depletion of substrates and rapid buildup of potentially toxic metabolites and is more suited to studying short-term community dynamics and assaying the end-products of fermentation. Chemostat bioreactors have been and continue to be successfully used to culture complex microbial communities found in feces^[28] ^and simpler defined bacterial communities^[29]^. These systems provide insight into compositional and metabolic changes in microbial communities over time and in response to perturbations^[30]^. A chemostat bioreactor with controlled inflow of fresh medium and outflow of waste, pH, or redox potential control is a more complicated method compared to batch culturing but allows the study of more complex microbial interactions. However, compared to batch experiments, the number of comparisons between conditions or inocula that can be made is limited. 

It is important to consider the growth rate of the microbes for the refreshing rate of the medium. Specifically, a constant flow of fresh nutrients can be applied, or pre-defined feed inputs at set times to mimic the host diet can be employed. For more high-throughput analyses, for example when multiple conditions need to be tested, mini-bioreactors can be applied^[31]^. Alternatively, a specific part of the gut can be modeled, such as the different regions of the intestinal tract, the mucus layer, or the lumen. For a more complete model of the intestinal tract, a more complex model such as mucosal-simulator of human intestinal microbial ecosystem (M-SHIME) can be applied. The M-SHIME model incorporates both a luminal and a mucosal compartment. Another example of a more complex model is the TNO *in vitro *model of the colon (TIM-2). TIM-2 models the colon by applying peristaltic movements and a dialysis system for the removal of metabolites^[32]^. Similarly, a recent system has been described to simulate the human ileum^[33]^. Furthermore, the SIMGI (simulator gastro-intestinal) model consists of a gastric component, a small intestine component, and three components that represent the different regions of the colon^[34]^.

The ultimate choice of which reactor set-up to apply for studying the synthetic microbial community of interest depends, among others, on the complexity of the community, the gut environment of interest, the research question(s), the desired output, and the availability of resources. For example, a batch culture system could be chosen when the impact of multiple food components is compared and contrasted, while a continuous culture setup would be the preferred choice when the response of the community after an intervention is the focus of the study.

### Deciding the parameters to analyze

It is crucial to define what output needs to be measured during growth of the synthetic microbial community to answer the research questions. This can include measurements of relative and/or absolute abundances, the decrease of substrate, the increase of metabolites, transcriptomics, and proteomics. Table 2 lists potential output of the synthetic community and examples of associated techniques.

**Table 2:** Potential output of a synthetic community and examples of tools that can be applied to measure these outputs

**Output**	**Technique**	**Advantages**	**Disadvantages**
Microbial composition and relative abundance	16S rRNA gene sequencing	Straightforward method and large database of sequences available allowing highly accurate comparisons	Only provides relative abundances*Species-level profiling not reliable
	Internal transcribed spacer sequencing	Species-level microbial profiling	Only provides relative abundances*
	Quantitative PCR	Provides relative and absolute abundances	Only possible when specific primers are available
Transcriptome	RNA sequencing	Not limited by the design of probes	Requires specific bioinformatic knowledge to handle large datasets
	Microarray	Examine the expression of thousands of genes simultaneously	Requires reference genomes for the organisms of interest to design probes
Proteome	Immunoassays (e.g., enzyme-linked immunosorbent assay)	Detection of proteins with a low abundance	Targets specific proteins
	Gel-based protein separation (e.g., sodium dodecyl sulfate-polyacrylamide gel electrophoresis)	Suitable for complex samples	Downstream analysis method requiredLabor intensive
	Chromatography-based protein separation	Suitable for complex samples	Downstream analysis method required
	Protein microarray	High throughputSuitable for complex samples	The antibodies need to be predefined
	Mass spectrometry	High throughputSensitiveSuitable for complex samples	Costly; specific analytical expertise needed
Metabolome/metabonome	High-performance liquid chromatography (HPLC)	Quick, automated, and accurate method to identify certain chemical components in a sample	Costly; several systems and columns needed for different chemical categories
	Liquid chromatography-mass spectrometry (LC-MS)	Separation of molecules (HPLC) combined with structural identification of individual compounds with high detection sensitivity and molecular specificity (MS)	Costly; not all mobile phases used for HPLC are compatible with MS
	Gas chromatography	Quick, automated, and accurate method to identify certain chemical components in a sample	Several systems and columns needed for different chemical categories
	*In vitro *nuclear magnetic resonance	Monitoring microbial metabolism; provides information about molecular structure at atomic level; pathway reconstruction	Only small molecules can be detected
Tracing substrate	DNA or RNA stable-isotope probing	Trace which community member incorporates a certain component	Careful experiment design, the required sensitivity, the approach selected for separating labeled nucleic acid biomarkers, and the downstream analysis intended for the labeled material
	*In vitro *nuclear magnetic resonance	Monitoring microbial metabolism; provides information about molecular structure at atomic level	Rather low sensitivity
	Bioorthogonal non-canonical amino acid tagging combined with fluorescently activated cell sorting and sequencing^[35]^	Identification of translationally active microorganisms in the community	New method, so requires further optimization and validation, in particular for application to synthetic microbial communities
Visualization	Fluorescence *in situ *hybridization (FISH)	Visualization of localization of community members	Limited by the availability of fluorescent probesNo quantificationDetection limit
	Microautoradioactivity combined with FISH	Identification of metabolically active microorganisms in mixed communities	Low rRNA copy numbers are undetectable with standard FISH techniques

*This can be overcome by adding an additional step for quantification, such as flow cytometry or quantitative PCR, to determine the total bacterial load^[36]^.

## CONCLUSION

Synthetic microbial communities are a great tool to study ecological interactions between gut microbes of interest. They can provide knowledge on microbial functionality beyond that derived from DNA-based techniques. Studies using synthetic communities have proven valuable for doing gut microbiota-related research in uncovering cross-feeding actions and collaborative resource sharing. For example, a bioreactor supplemented with dietary fibers and mucin was applied to study trophic interactions and niche occupation between microbes *in vitro*^[23]^. The application of synthetic microbial communities still has several limitations. First, not all gut microbes have been cultured or have their genome sequence determined, which could result in a lack of function in the synthetic community. Second, even though synthetic communities are designed to mimic the natural environment as well as possible, microbes could adopt different roles than the ones they have *in vivo*. As such, host factors such as hormones and immune factors are usually either not or poorly represented in *in vitro *models. Third, tracing molecules in a synthetic community is difficult, as it is not possible to distinguish which species are using a certain substrate, or which species produce a certain metabolite.

However, to move forward and provide functional data on microbial ecosystems, we are convinced that synthetic communities are the way to go, and this review provides a workflow on how to conduct qualitative experiments when doing so. We provide examples of how a synthetic minimal microbiome can be applied to answer research questions of interest. We see great potential, for instance when the goal of the research is to achieve a certain functional output, such as the one described for optimizing butyrate production^[18]^. Metabolic modeling can aid the design of synthetic communities, as metabolic models can predict functions, interactions, and responses to condition changes, but also point towards knowledge gaps. Therefore, *in vitro *synthetic communities and metabolic modeling can complement each other^[37,38]^. Future research on synthetic communities for modeling the gut microbiota holds great potential for new insight into microbial actions and functionality that impact the gut system. Moreover, advances in synthetic biology will certainly help in investigating yet unknown gut microbial functions observed by metagenomics^[39]^.

## DECLARATIONS

### Copyright

© The Author(s) 2022.
